# Combined DTI Tractography and Functional MRI Study of the Language Connectome in Healthy Volunteers: Extensive Mapping of White Matter Fascicles and Cortical Activations

**DOI:** 10.1371/journal.pone.0152614

**Published:** 2016-03-30

**Authors:** François Vassal, Fabien Schneider, Claire Boutet, Betty Jean, Anna Sontheimer, Jean-Jacques Lemaire

**Affiliations:** 1 IGCNC *(Image-Guided Clinical Neuroscience and Connectomics)*, EA 7282, Unité de Formation et de Recherche Médecine, Université d’Auvergne, Clermont-Ferrand, France; 2 Service de Neurochirurgie, Hôpital Nord, Centre Hospitalier Universitaire de Saint-Etienne, Saint-Etienne, France; 3 Unité de Neuroradiologie, Hôpital Nord, Centre Hospitalier Universitaire de Saint-Etienne, Saint-Etienne, France; 4 Unité de Neuroradiologie, Hôpital Gabriel Montpied, Centre Hospitalier Universitaire de Clermont-Ferrand, Clermont-Ferrand, France; 5 Service de Neurochirurgie, Hôpital Gabriel Montpied, Centre Hospitalier Universitaire de Clermont-Ferrand, Clermont-Ferrand, France; Institute of Psychology, Chinese Academy of Sciences, CHINA

## Abstract

Despite a better understanding of brain language organization into large-scale cortical networks, the underlying white matter (WM) connectivity is still not mastered. Here we combined diffusion tensor imaging (DTI) fiber tracking (FT) and language functional magnetic resonance imaging (fMRI) in twenty healthy subjects to gain new insights into the macroscopic structural connectivity of language. Eight putative WM fascicles for language were probed using a deterministic DTI-FT technique: the arcuate fascicle (AF), superior longitudinal fascicle (SLF), uncinate fascicle (UF), temporo-occipital fascicle, inferior fronto-occipital fascicle (IFOF), middle longitudinal fascicle (MdLF), frontal aslant fascicle and operculopremotor fascicle. Specific measurements (i.e. volume, length, fractional anisotropy) and precise cortical terminations were derived for each WM fascicle within both hemispheres. Connections between these WM fascicles and fMRI activations were studied to determine which WM fascicles are related to language. WM fascicle volumes showed asymmetries: leftward for the AF, temporoparietal segment of SLF and UF, and rightward for the frontoparietal segment of the SLF. The lateralization of the AF, IFOF and MdLF extended to differences in patterns of anatomical connections, which may relate to specific hemispheric abilities. The leftward asymmetry of the AF was correlated to the leftward asymmetry of fMRI activations, suggesting that the lateralization of the AF is a structural substrate of hemispheric language dominance. We found consistent connections between fMRI activations and terminations of the eight WM fascicles, providing a detailed description of the language connectome. WM fascicle terminations were also observed beyond fMRI-confirmed language areas and reached numerous cortical areas involved in different functional brain networks. These findings suggest that the reported WM fascicles are not exclusively involved in language and might be related to other cognitive functions such as visual recognition, spatial attention, executive functions, memory, and processing of emotional and behavioral aspects.

## Introduction

Seminal lesion studies in aphasiology [1] have provided a topological model of brain language organization where three cortical territories, i.e. Broca’s, Geschwind’s and Wernicke’s, play central roles in language production and comprehension. In the past two decades, functional magnetic resonance imaging (fMRI) has changed our understanding of language organization into a network model by showing activations within a distributed set of regions that extend beyond these three cortical territories [2–5]. In this framework, language is underpinned by large-scale neuronal networks that co-interact in what is known as the language connectome [6]. These networks rely on anatomical connections between language-related brain regions. These connections are established by white matter (WM) fascicles, which represent the macroscopic organization of densely-packed and roughly parallel groups of axons [7] connecting structurally and functionally remote brain regions. The cortical regions involved in language processing have been widely explored with fMRI [2–5] and cortical intraoperative electrical stimulation (IES) [8–10], yet the underlying subcortical connectivity is still not extensively mastered. Studies using diffusion tensor imaging (DTI) fiber tracking (FT) [6,11–17] and subcortical IES [18] have produced a lot of data but no firm conclusions on the precise anatomy and functionality of language-related WM fascicles (see, e.g., [19] for a recent review). In particular, although the functional roles of a WM fascicle could be inferred from its topography within the brain [11], the cortical terminations are still not firmly established (see S1 Table). There is growing consensus that WM fascicles classically considered language-specific are in fact “multi-function” rather than specialized, such the arcuate fascicle that processes non-linguistic sound localization and auditory spatial awareness [20]. Deeper knowledge of the anatomy of WM fascicles could therefore yield critical insights into language and other cognitive skills.

We hypothesized that combining language fMRI and DTI-FT could deliver new insights into the macroscopic structural organization of the language connectome and cortical terminations of WM fascicles. Cortical language regions were explored by measuring modifications in blood-oxygen-level-dependent (BOLD) signal using a specific task involving phonological, semantic and syntactic information to cover a broad spectrum of language processing. In parallel, eight WM fascicles that have been proposed to support language were probed using a deterministic DTI-FT technique and seeded from regions of interest (ROIs) placed manually within the WM. Morphological and biophysical parameters were explored for each WM fascicle, i.e. volume, length, fractional anisotropy (FA) and left–right asymmetries. We analyzed the cortical terminations of each of the right and left WM fascicles within the frontal, parietal, temporal and occipital lobes. We also tracked the connections between WM fascicles and BOLD clusters to figure out which WM fascicles are related to language processing. The aim of using this construct was to define in explicit detail the language connectome and the precise and complete cortical terminations of WM fascicles.

## Materials and Methods

### Subjects

The study enrolled 20 healthy native-French-speaking subjects (mean age = 25 ± 5 [19–40] years). All participants were medical students or hospital engineers. Absence of language disorders was checked prior to study enrollment. Recruitment was restricted to right-handed male subjects (Edinburgh handedness inventory [21]) as handedness and gender have been shown to influence language lateralization [22]. Approval was obtained from the local Research Ethics Committee (CPP Sud-Est VI; approval number AU 1061) and written informed consent was obtained from all subjects prior to enrollment.

### MRI Acquisition

MRI studies were performed on a 3-Tesla machine (GE Discovery MR750, General Electric, Milwaukee, WI) using a 32-channel head coil. Whole-brain high-resolution T1-weighted images for anatomical registration were acquired with a three-dimensional (3D) inversion recovery gradient-echo sequence (BRAVO), yielding 288 interleaved slices of 1.4-mm thickness in the axial plane: TR = 8.8 s, TE = 3.6 ms, TI = 400 ms, flip angle = 12°, FOV = 240 mm, matrix = 510×510, resulting voxel size = 0.47×0.47×0.7 mm^3^. For the language fMRI experiment, single-shot gradient-echo echo-planar images were acquired to provide BOLD contrast: TR = 3 s, TE = 30 ms, flip angle = 90°, FOV = 240 mm, matrix = 64×64, 48 contiguous axial slices of 4-mm thickness to cover the whole brain, resulting voxel size = 3.7×3.75×4 mm^3^. The single-shot spin-echo echo-planar sequence was used for DTI with diffusion gradients along 20 directions: b-value = 0 and 1,000 mm^2^/s, TR = 7 s, TE = 86 ms, FOV = 280 mm, matrix = 256×256, 46 contiguous axial slices of 3.5-mm thickness to cover the whole brain, resulting voxel size = 1×1×3.5 mm^3^.

### fMRI Paradigm

Statistical maps of fMRI BOLD signal were created using a block experimental design. BOLD contrast measurements were performed with cycles of 22 s “on” task (language task) and 22 s “off” task (control task) repeated 10 times, resulting in a total presentation time of 7 min 20 s. To assess the main components of language, we used an alternating sentence- and letter-decision task relying on visual stimuli; this experimental design provides robust activations covering all essential language areas in both healthy subjects and patients [23,24], and is correlated with the Wada test in preoperative determination of hemispheric language dominance [23]. In the sentence-decision task (activation condition) engaging visual, phonological, semantic and syntactic information processing, subjects determined whether pairs of grammatically different sentences contained the same meaning. In the letter-decision task (baseline condition) designed to control for visual input, subjects were asked whether pairs of non-pronounceable consonant strings were identical. All participants were given pre-training to perform the language and control tasks accurately. Error scores were not monitored during the fMRI.

Both tasks consisted of pairs of items, with half of the trials requiring a correct response and half requiring an incorrect response. The pairs of items were projected in a white boldface font on a black background, one item above the other. For the sentence task, pairs of grammatically-varied sentences with the same content words were constructed to hold either the same meaning (e.g. “the fighter plane destroyed the tank” and “the tank has been destroyed by the fighter plane”) or a different meaning (e.g. “the little boy is smiling at the schoolgirl with the black book bag” and “the schoolgirl who is smiling at the little boy has a black book bag”) meaning. Grammatical variations revolved around active/passive voice. All sentences had the same simple underlying subject–verb–object structure and were reversible, i.e. the object could just as plausibly be the subject of the action. For the letter task, pairs of consonant-strings (each string consisting of 6 different consonants randomly chosen from the alphabet) were constructed as either identical (e.g. “Ghrwst” and “Ghrwst”) or different (e.g. “Tmqvbd” and “Tmkvbd”) by just one consonant. We assumed that subtracting the letter from the sentence task would make it possible to isolate the language regions of interest associated with phonological, semantic and syntactic processing, except for regions specifically involved in visual letter recognition.

Functional maps of BOLD signal were computed using Statistical Parametric Mapping (SPM8, Wellcome Trust Centre for Neuroimaging, University College London, UK) run in MATLAB R2012a (MathWorks, Natick, MA). For first-level single-subject analysis, functional images were preprocessed for slice timing and motion corrections, and smoothed with a 6×6×6 3D Gaussian kernel [25]. For second-level group analysis, anatomical and functional volumes were co-registered, spatially normalized into the Montreal Neurological Institute (MNI) template, and the preprocessed functional images were smoothed with an 8×8×8 3D Gaussian kernel. Voxel-wise analysis of BOLD signal consisted of modeling the activation and baseline conditions with a boxcar function convolved with the canonical hemodynamic response function using a general linear model [26] with motion parameters as covariates and applying a 128 Hz high-pass filter [27]. fMRI contrast analysis used a one-sample *t-*test, at a statistical threshold set at p < 0.05, with family-wise error correction for the whole brain and a minimum cluster extent of two contiguous voxels (k = 2).

### BOLD-Cluster Maps

**Fig 1:**
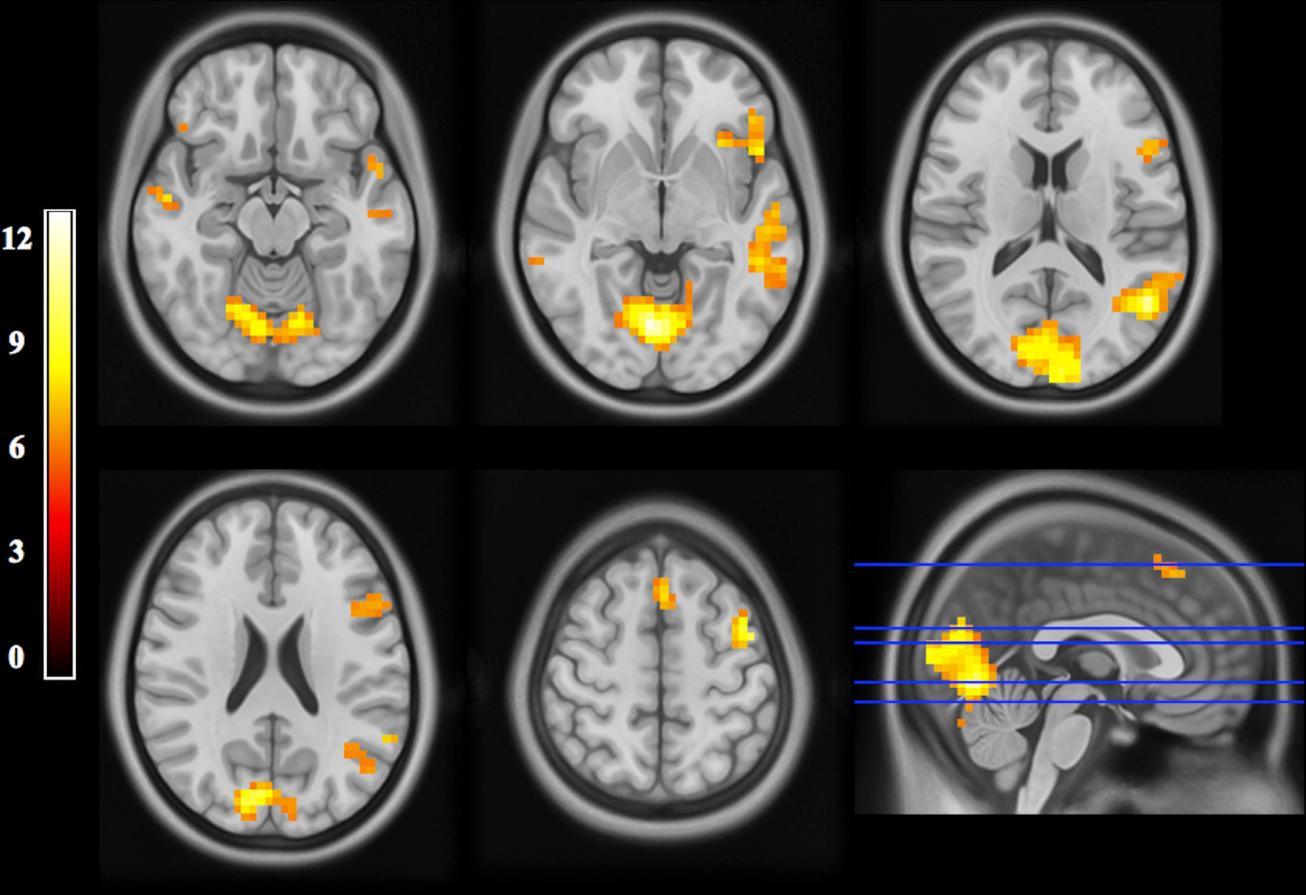
fMRI maps (group analysis). Statistical maps (axial slices oriented in radiological convention, i.e. where the left side of the images corresponds to the right side of the brain; MNI coordinates, z = -15, -5, 15, 23, 55) of fMRI BOLD signal following a sentence-decision task (see text for details): group analysis of 20 healthy subjects (SPM8; statistical threshold set at p < 0.05, with family-wise error correction; cluster > 2 contiguous voxels). Cortical areas of activations are visible within the primary/secondary visual cortex, middle/superior temporal gyrus, inferior frontal gyrus and supplementary motor area of the left and right hemispheres, as well as within the temporal pole, inferior parietal lobule, premotor cortex and middle frontal gyrus of the left hemisphere.

The fMRI experiment resulted in BOLD-cluster maps showing known language areas (Fig 1, Table 1 and S2 Table): the supplementary motor area (SMA; dorsal part of Brodmann area [BA] 6); posterior part of the middle frontal gyrus (pMFG; BA 9); inferior frontal gyrus (IFG) or Broca’s area (pars opercularis, triangularis and orbitalis of the IFG, BA 44/45/47); inferior parietal lobule (IPL) or Geschwind’s area, including the supramarginal (SMG, BA 40) and angular (AG, BA 39) gyrus; posterior part of the middle (pMTG, BA 20) and superior (pSTG, BA 21) temporal gyrus or Wernicke’s area; and temporal pole (TP, BA 38).

**Table 1:** Distribution of BOLD clusters (group analysis).

Anatomical labeling of activation areas (AAL atlas)	BA	Cluster size (voxels)	Peak coordinates (MNI template)			Maximum *t* value
Left MTG, STG	21/22	230	-50	-60	14	11.19
Left IFG (pars opercularis, triangularis, orbitalis), vPMC	44/45/47/6	133	-46	32	-2	9.68
Left dPMC, MFG	6/9	42	-42	0	58	10.98
Left, Right SMA	6	32	-2	20	58	8.62
Left AG	39	10	-38	-63	43	7.66
Left SMG	40	8	-58	-52	30	7.75
Left TP	38	8	-49	13	-14	7.84
Right aMTG	21	8	54	-8	-14	8.02
Right pMTG	22	5	62	-40	-6	6.67
Right IFG (pars orbitalis)	47	6	42	28	-10	6.94
Right IFG (pars triangularis)	45	5	54	28	6	6.87
Right IFG (pars opercularis)	44	5	65	13	7	6.77
Left, Right occipital lobe (CN, LG, MOG, SOG)	17/18/19	405	-6	-96	10	12.77
Left, Right cerebellum		290	18	-72	-34	12.33

Areas of activation following a sentence-decision task (see text for details): group analysis of 20 healthy subjects (SPM8; statistical threshold set at p < 0.05, with family-wise error correction; cluster > 2 contiguous voxels). AAL = automated anatomical labeling; AG = angular gyrus; aMTG = anterior part of the middle temporal gyrus; BA = Brodmann area; CN = cuneus; dPMC = dorsal premotor cortex; IFG = inferior frontal gyrus; LG = lingual gyrus; MFG = middle frontal gyrus; MNI = Montreal Neurological Institute; MOG = middle occipital gyrus; pMTG = posterior part of the middle temporal gyrus; SMA = supplementary motor area; SMG = supramarginal gyrus; SOG = superior occipital gyrus; STG = superior temporal gyrus; TP = temporal pole; vPMC = ventral premotor cortex.

### Tractography

Eight WM fascicles that have been proposed to support language [6,11–19] were explored (see S1 Glossary): (1) the arcuate fascicle (AF); (2) the frontoparietal (SLF-fp) and temporoparietal (SLF-tp) segments of the superior longitudinal fascicle part III; (3) the uncinate fascicle (UF); (4) the temporo-occipital fascicle (TOF); (5) the inferior fronto-occipital fascicle (IFOF); (6) the middle longitudinal fascicle (MdLF); (7) the frontal aslant fascicle (FAF); and (8) the operculopremotor fascicle (OpPMF).

All FT procedures were performed by a trained clinical neuroanatomist (F.V.) using fiber assignment by continuous tracking (FACT) [28] and tensor deflection (TEND) [29] methods on iPlan Stereotaxy 3.0 software (BrainLab, Feldkirchen, Germany). FA threshold for FT was set to 0.20. Note that amount of new insight gainable when investigating the anatomy of WM fascicles depends on the extent to which reconstruction of the fibers is constrained *a priori* by ROI positioning. As the goal of our study was a precise and extended cortical terminations map, seed ROIs were placed in WM regions of “obligatory passages” along the path of each WM fascicle [11,30,31], i.e. we did not use target ROIs in *a priori* terminating cortical regions. This made it possible to visualize all streamlines of a single WM fascicle without constraining its cortical terminations, which can vary hemisphere-to-hemisphere and subject-to-subject. Briefly, each WM fascicle was generated as follows: (i) guided by color-coded direction maps, positioning of two seed ROIs on FA maps within WM voxels where the fascicle’s fibers were easily identifiable; (ii) iterative test-retests, changing the size and shape of the ROIs, checking that no fibers belonging to the fascicle were missed; (iii) use of a “fiber exclusion” tool (iPlan Stereotaxy 3.0) when the on-line reconstruction produced fibers obviously not included in the fascicle (e.g. presence of a frontoparietal fiber when tracking the AF); (iv) generation of a 3D object for each fascicle, ready for further analysis of the whole macroscopic structural organization.

### Quantitative Analysis of WM Fascicles

For each WM fascicle, we measured volume, length and mean FA and calculated a lateralization index (LI) according to the following formula (e.g. for volume): (volume Left—volume Right) / (volume Left + volume Right) (between +1 and -1, where a positive value means leftward and a negative value means rightward) [32]. The statistical significance of degree of lateralization was determined using a one-sample *t*-test (normal distribution checked, significance threshold set to p < 0.05) adjusted for multiple comparisons with Bonferonni correction. We tested for significant lateralization of WM fascicle volumes across all 20 subjects. Length and mean FA, which reflect specificity and entirety of the underlying fiber tract micro-anatomic architecture, were also analyzed to evaluate FT reproducibility; we hypothesized that length and mean FA would be similar within both hemispheres.

### Cortical Terminations of WM Fascicles

**Fig 2:**
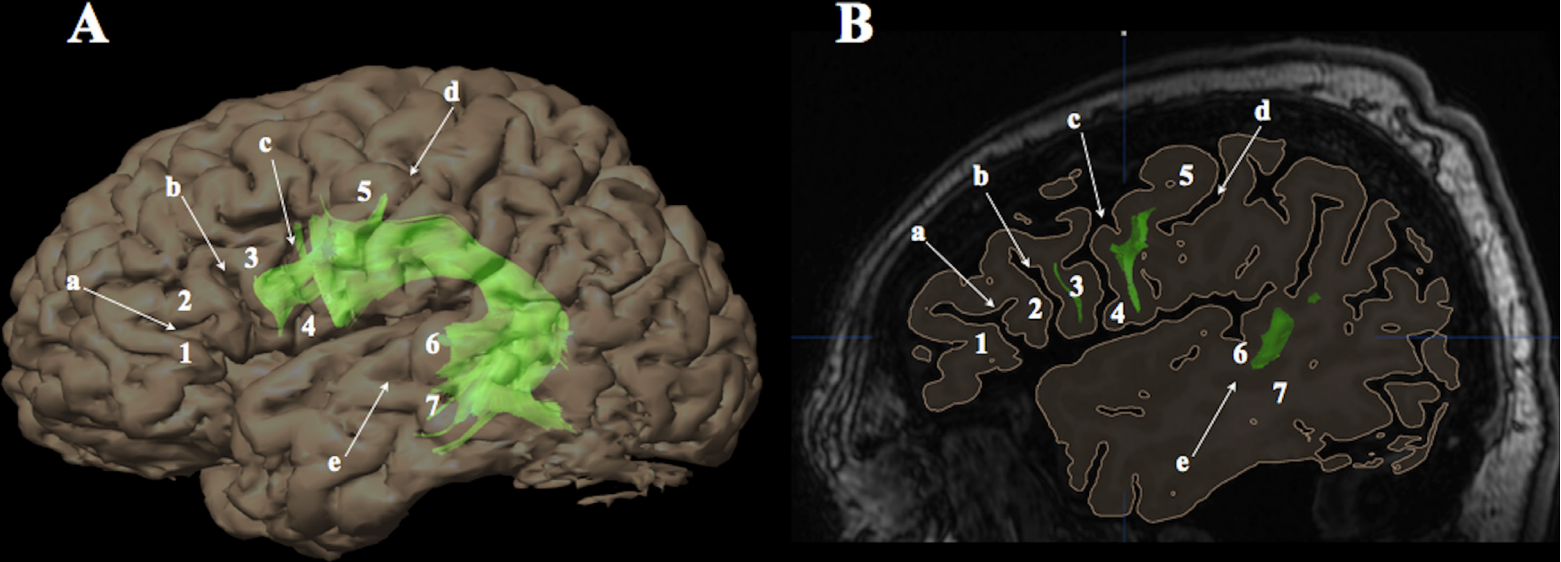
Labeling of cortical terminations according to anatomical landmarks. Cortical terminations of white matter fascicles (e.g. left arcuate fascicle; green) were labeled according to gyri and sulci provided by 3D surface renderings (A) generated from high-resolution T1-weighted magnetic resonance images (B; sagittal section running through the left frontal operculum). a = horizontal ramus of the Sylvian fissure; b = ascending ramus of the Sylvian fissure; c = precentral sulcus; d = central sulcus; e = superior temporal sulcus; 1 = pars orbitalis of the inferior frontal gyrus (Brodmann area [BA] 47); 2 = pars triangularis (BA 45); 3 = pars opercularis (BA 44); 4 = ventral premotor cortex (BA 6); 5 = precentral gyrus (BA 4/6); 6 = superior temporal gyrus (BA 22); 7 = middle temporal gyrus (BA 21).

Image datasets were co-registered (mutual information algorithm) using iPlan Stereotaxy 3.0 software. The FA, color-coded direction, and BOLD-cluster maps were co-registered with T1-weighted MR images (anatomical reference). Accuracy of registration was carefully reviewed (visual analysis of merged images and test-retests) using the following landmarks: putamen, pallidum, corpus callosum (whole body and major and minor forceps), anterior and posterior limbs of the internal capsule, cerebellar contour, tentorium of the posterior fossa, sylvian region, upper brainstem contour, ventricular system (frontal horns and trigone), interhemispheric fissure and main cerebral gyrations. For each individual, we generated 3D surface renderings of the brain from T1-weighted MR images used to determine the cortical terminations of WM fascicles within the frontal, parietal, temporal and occipital lobes of the right and left hemispheres. Cortical territories were labeled according to classical anatomical terminology, as follows (Fig 2): frontal pole (FP, BA 10); lateral (lOrbF, BA 11) and medial (mOrbF, BA 12) orbitofrontal cortex; subgenual cortex (SubG, BA 25); SMA (dorsal part of BA 6); MFG (BA 9/46); Broca’s area, located in the pars opercularis, triangularis and orbitalis of the IFG (BA 44/45/47); vPMC (ventral part of BA 6); superior parietal lobule (SPL, BA 7); precuneus (PCN, BA 7); Geschwind’s area, located in the IPL including the AG (BA 39) and SMG (BA 40) gyrus; TP (BA 38); parahippocampal gyrus (PHG, BA 28); uncus (UNC, BA 34); Wernicke’s area, located in the pMTG/pSTG (BA 21/22); inferior temporal gyrus (ITG, BA 20); temporo-occipital cortex including the fusiform gyrus (T-O, BA 37); and occipital lobe (OL) including cuneus (CN), lingual gyrus and lateral occipital gyri (BA 18/19).

### Connections between WM Fascicles and BOLD Clusters

**Fig 3:**
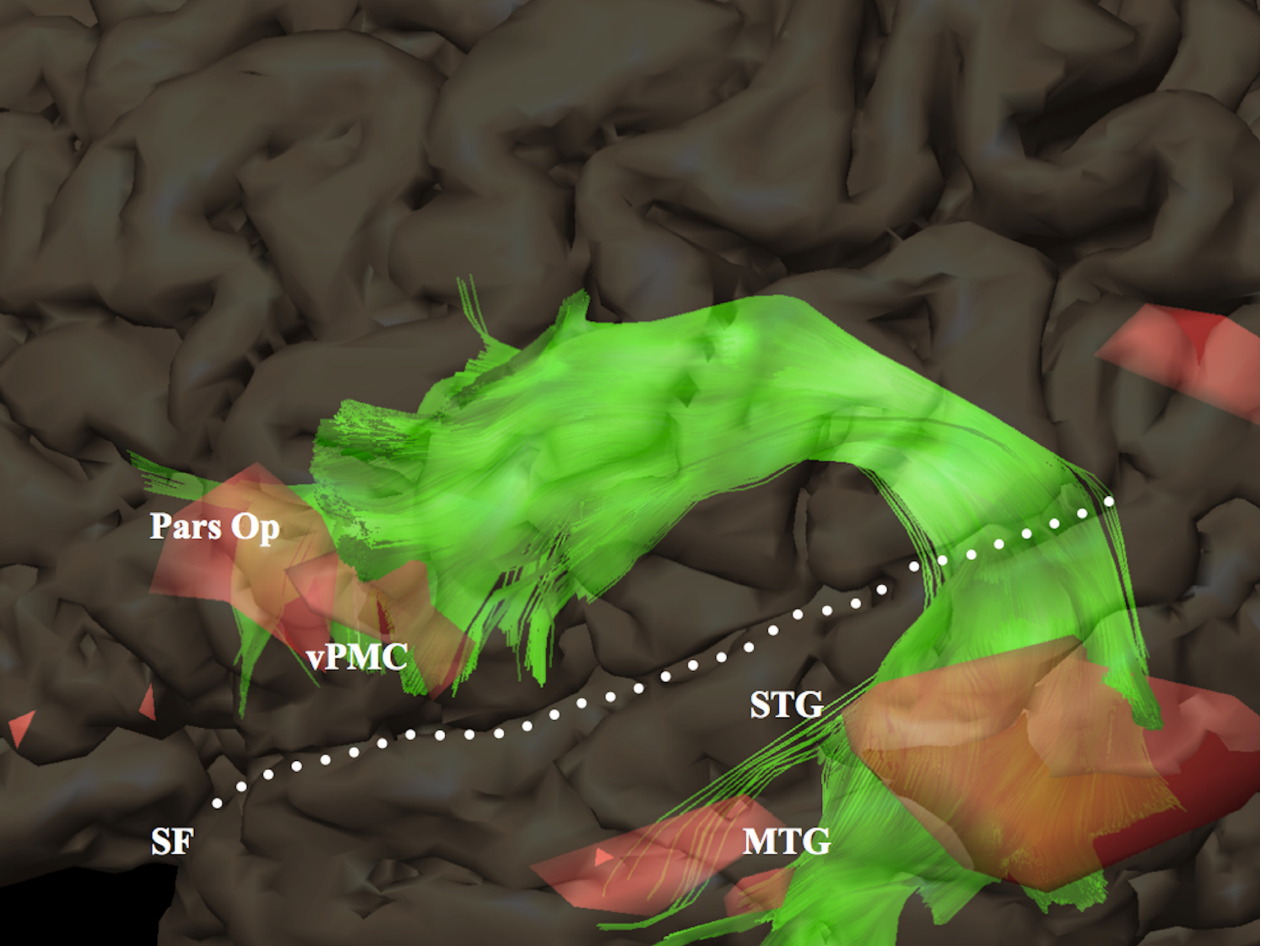
Example of white matter fascicle–BOLD cluster connections (same subject as in Fig 2). Zoom-in showing a 3D rendering of BOLD clusters (red): terminations of the left arcuate fascicle (green) go through BOLD clusters located within the posterior part of the middle and superior temporal gyrus, ventral premotor cortex and pars opercularis of the inferior frontal gyrus. MTG = middle temporal gyrus; Pars Op = pars opercularis; SF = Sylvian fissure; STG = superior temporal gyrus; vPMC = ventral premotor cortex.

Structure–function relationships were investigated by analyzing WM fascicle–BOLD cluster connections at individual level (each subject being its own anatomical reference) within cortical territories known as essential language areas in order to figure out which WM fascicles are related to language processing. A connection was determined when fibers went through a cluster, regardless of number of fibers involved (Fig 3). Since fMRI clusters were located within the gray matter and fascicles terminated in the subcortical WM, each cluster was enlarged by 4 mm in the depth direction using a “scaling object” tool (iPlan Stereotaxy 3.0) to encompass the gray–white matter interface.

### Correlation between Lateralization Indexes of WM Fascicles and BOLD Clusters

Structure–function relationships were also investigated by looking for correlation between the LIs of WM fascicles and BOLD clusters: LI of WM fascicles was calculated from the right and left volumes (see above); LI of BOLD clusters was defined as the ratio (L–R) / (L + R), where L and R are number of activated voxels in the left and right hemisphere, respectively, yielding scores on a gradient from +1 to -1 where positive means left- and negative right-hemispheric language dominance [33]. Pearson’s correlation coefficients (normal distribution checked) were computed to test the hypothesis that LIs of WM fascicles would correlate with BOLD clusters, e.g. subjects with thicker WM fascicles in the left hemisphere would also having more left-lateralized language functions.

## Results

### Quantitative Data on WM Fascicles

We found a leftward structural asymmetry (i.e. greater volume on the left) of the AF (p = 0.000004), SLF-tp (p = 0.0001) and UF (p = 0.0004), whereas SLF-fp was thicker (p = 0.0001) within the right hemisphere. We did not find left–right differences in length and mean FA. Volume, length and mean FA of the left and right WM fascicles are summarized in Table 2.

**Table 2:** Characteristics of the white matter fascicles.

WM fascicles	Left hemisphere			Right hemisphere			Lateralization index		
	Volume	FA	Length	Volume	FA	Length	Volume	FA	Length
**AF**	12.2±4.5	0.47±0.02	15.3±2.9	7.4±2.9	0.48±0.02	13.7±2.6	0.26±0.19*	-0.01±0.03	0.02±0.08
**SLF-fp**	5.1±1.7	0.42±0.03	9.3±1.5	9.5±2.4	0.44±0.02	9.7±2.2	-0.14±0.12*	-0.02±0.04	-0.05±0.12
**SLF-tp**	8.8±2.6	0.42±0.02	8.3±0.79	5.8±2.4	0.43±0.03	7.8±1.1	0.22±0.20*	-0.01±0.05	0.07±0.11
**UF**	10.1±3.3	0.42±0.03	12.9±1.5	7.5±1.3	0.42±0.02	12.2±1.4	0.13±0.12*	-0.003±0.04	0.05±0.09
**TOF**	11.6±3.4	0.45±0.02	13.6±2.6	10.1±3.3	0.46±0.02	13.5±2.9	0.09±0.12	-0.02±0.06	0.02±0.04
**IFOF**	17.4±3.8	0.47±0.02	21.6±2.1	16.1±3.5	0.50±0.02	20.5±3.2	0.05±0.14	-0.002±0.03	0.04±0.04
**MdLF**	6.5±2.1	0.41±0.04	13.4±2.8	6.3±2.0	0.43±0.03	13.2±1.8	0.02±0.21	-0.005±0.03	-0.004±0.13
**FAF**	6.6±2.2	0.39±0.03	8.1±1.1	6.3±2.9	0.39±0.02	8.5±0.9	0.05±0.03	0.012±0.03	-0.002±0.04
**OpPMF**	2.7±1.2	0.39±0.04	6.5±1.5	2.5±1.2	0.40±0.02	6.6±1.2	0.10±0.35	-0.01±0.03	0.03±0.16

Means and standard deviations of volume (cm^3^), FA, length (cm) and lateralization index (see text for details) of the eight white matter fascicles systematically identified within the left and right hemispheres (20 healthy subjects). AF = arcuate fascicle; FA = fractional anisotropy; FAF = frontal aslant fascicle; IFOF = inferior fronto-occipital fascicle; MdLF = middle longitudinal fascicle; OpPMF = operculopremotor fascicle; SLF-fp = frontoparietal segment of the superior longitudinal fascicle; SLF-tp = temporoparietal segment of the superior longitudinal fascicle; TOF = temporo-occipital fascicle; UF = uncinate fascicle; WM = white matter.

* p < 0.05 after Bonferroni correction.

### Cortical Terminations of WM Fascicles

**Fig 4:**
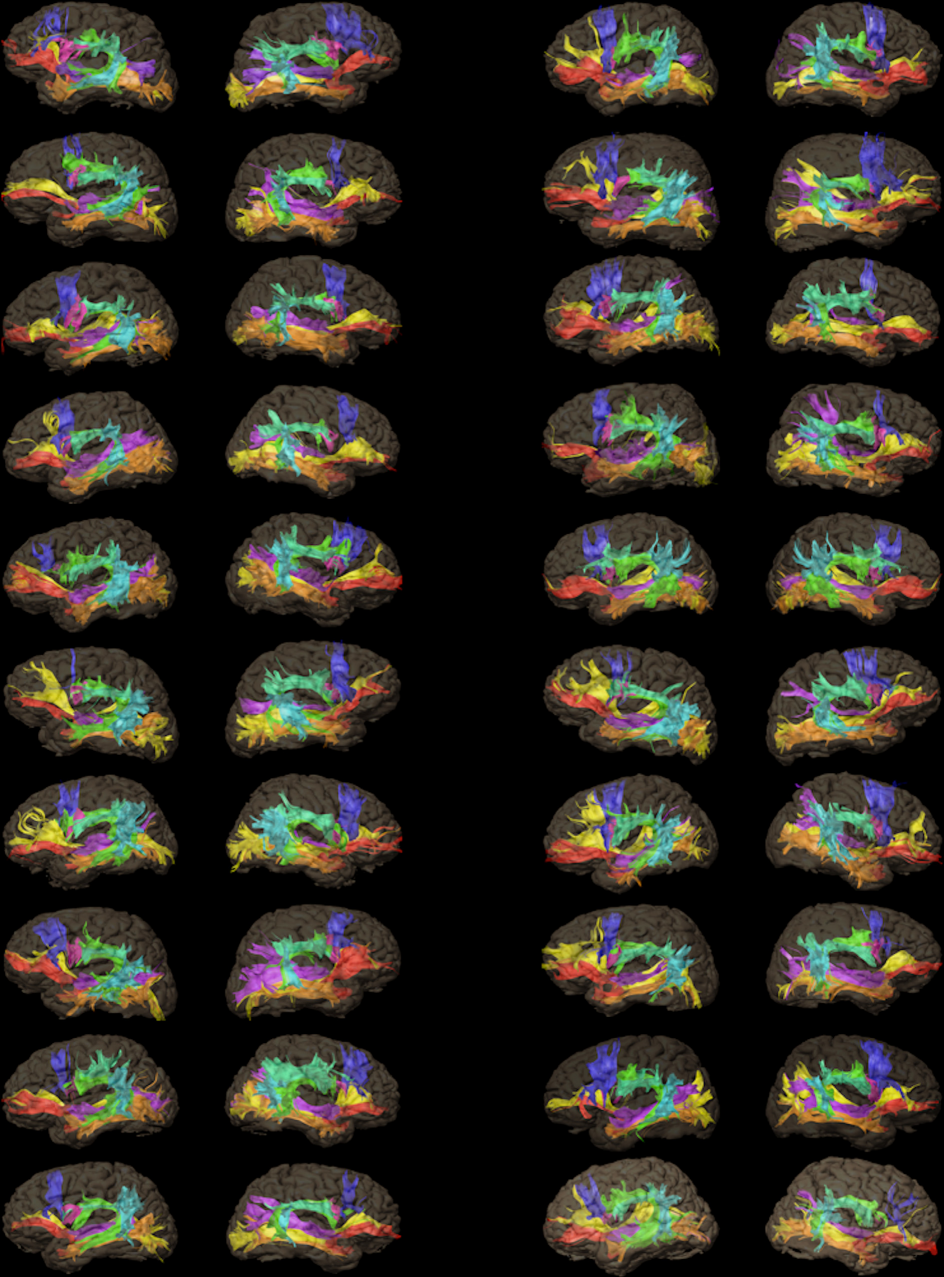
Overall display of the white matter fascicles. Display of the eight white matter fascicles systematically reconstructed within the left (left column) and right (right column) hemispheres (20 healthy subjects). Color code: green = arcuate fascicle; turquoise = frontoparietal segment of the superior longitudinal fascicle; light blue = temporoparietal segment of the superior longitudinal fascicle; red = uncinate fascicle; orange = temporo-occipital fascicle; yellow = inferior fronto-occipital fascicle; purple = middle longitudinal fascicle; blue = frontal aslant fascicle; pink = operculopremotor fascicle.

We successfully reconstructed the eight WM fascicles bilaterally in all 20 healthy subjects, thus giving a total of 320 WM fascicles (Fig 4). The cortical terminations of each WM fascicle within the left and right hemispheres are reported in Table 3 along with their percent occurrences. Broadly, we observed an asymmetrical representation of WM fascicles, with different connection patterns (i.e. whole cortical territories reached by the fiber terminations) within the left and right hemispheres (intra-subject variability) as well as between subjects. The most frequent connection patterns are reported in Table 4. Three WM fascicles showed standout asymmetries: (1) AF showed predominant direct connections between Broca’s and Wernicke’s areas in the left hemisphere (100% of subjects) but not in the right (only 40%); (2) IFOF showed prominent connections with the SPL in the right hemisphere (45% of subjects) but not in the left (0%); (3) MdLF showed prominent connections with the SPL in the right hemisphere (55% of subjects) but not in the left (only 15%), and connections with the AG were more prevalent in the left hemisphere (95%) than the right (65%). The whole-brain connectivity of the eight WM fascicles is further discussed in S1 Text.

**Table 3:** Cortical terminations of white matter fascicles.

Cortical territories	AF		SLF-fp		SLF-tp		UF		TOF		IFOF		MdLF		FAF		OpPMF	
	LH	RH	LH	RH	LH	RH	LH	RH	LH	RH	LH	RH	LH	RH	LH	RH	LH	RH
**SMA (BA 6)**	—	—	—	—	—	—	—	—	—	—	—	—	—	—	1.0 (20)	1.0 (20)	—	—
**MFG (BA 9/46)**	0.30 (6)	0.15 (3)	—	—	—	—	—	—	—	—	0.70 (14)	0.35 (7)	—	—	—	—	—	—
**FP (BA 10)**	—	—	—	—	—	—	0.55 (11)	0.50 (10)	—	—	1.0 (20)	1.0 (20)	—	—	—	—	—	—
**lOrbF (BA 11)**	—	—	—	—	—	—	1.0 (20)	1.0 (20)	—	—	1.0 (20)	1.0 (20)	—	—	—	—	—	—
**mOrbF (BA 12)**	—	—	—	—	—	—	0.50 (10)	0 .50 (10)	—	—	0.80 (16)	0.80 (16)	—	—	—	—	—	—
**SubG (BA 25)**	—	—	—	—	—	—	0.60 (12)	0.50 (10)	—	—	—	—	—	—	—	—	—	—
**Pars Orb (BA 47)**	—	—	—	—	—	—	0.65 (13)	0.55 (11)	—	—	0.80 (16)	0.65 (13)	—	—	—	—	—	—
**Pars Tr (BA 45)**	0.35 (7)	0.05 (1)	0.05 (1)	0.05 (1)	—	—	0.15 (3)	0.10 (2)	—	—	0.40 (8)	0.20 (4)	—	—	0.95 (19)	0.95 (19)	—	—
**Pars Op (BA 44)**	0.95 (19)	0.40 (8)	0.40 (8)	0.35 (7)	—	—	—	—	—	—	—	—	—	—	1.0 (20)	1.0 (20)	1.0 (20)	1.0 (20)
**vPMC (BA 6)**	1.0 (20)	1.0 (20)	1.0 (20)	1.0 (20)	—	—	—	—	—	—	—	—	—	—	1.0 (20)	1.0 (20)	1.0 (20)	1.0 (20)
**SMG (BA 40)**	—	—	1.0 (20)	1.0 (20)	0.45 (9)	0.45 (9)	—	—	—	—	—	—	0.10 (2)	—	—	—	—	—
**AG (BA 39)**	—	—	—	—	1.0 (20)	1.0 (20)	—	—	—	—	—	—	0.95 (19)	0.65 (13)	—	—	—	—
**SPL (BA 7)**	—	—	—	—	—	—	—	—	—	—	—	0.45 (9)	0.15 (3)	0.55 (11)	—	—	—	—
**PCN (BA 7)**	—	—	—	—	—	—	—	—	—	—	0.35 (7)	0.80 (16)	0.10 (2)	0.25 (5)	—	—	—	—
**OL (BA 18/19)**	—	—	—	—	—	—	—	—	1.0 (20)	1.0 (20)	1.0 (20)	1.0 (20)	0.55 (11)	0.55 (11)	—	—	—	—
**T-O (BA 37)**	—	—	—	—	—	—	—	—	1.0 (20)	1.0 (20)	0.90 (18)	0.90 (18)	0.10 (2)	0.10 (2)	—	—	—	—
**STG (BA 22)**	1.0 (20)	0.80 (16)	—	—	0.65 (13)	0.75 (15)	—	—	—	—	—	—	1.0 (20)	1.0 (20)	—	—	—	—
**MTG (BA 21)**	1.0 (20)	1.0 (20)	—	—	1.0 (20)	0.75 (15)	—	—	1.0 (20)	1.0 (20)	0.90 (18)	0.90 (18)	—	—	—	—	—	—
**ITG (BA 20)**	—	—	—	—	—	—	—	—	1.0 (20)	1.0 (20)	0.85 (17)	0.85 (17)	—	—	—	—	—	—
**UNC (BA 35)**	—	—	—	—	—	—	0.95 (19)	0.90 (18)	—	—	—	—	—	—	—	—	—	—
**PHG (BA 28)**	—	—	—	—	—	—	0.90 (18)	0.90 (18)	—	—	—	—	—	—	—	—	—	—
**TP (BA 38)**	—	—	—	—	—	—	1.0 (20)	1.0 (20)	1.0 (20)	1.0 (20)	—	—	0.90 (18)	0.90 (18)	—	—	—	—

Occurrence (percentage; number in brackets) of the terminations of the eight WM fascicles, according to cortical territories (20 healthy subjects). AF = arcuate fascicle; AG = angular gyrus; BA = Brodmann area; FAF = frontal aslant fascicle; FP = frontal pole; IFOF = inferior fronto-occipital fascicle; ITG = inferior temporal gyrus; LH = left hemisphere; lOrbF = lateral orbitofrontal cortex; MdLF = middle longitudinal fascicle; MFG = middle frontal gyrus; mOrbF = medial orbitofrontal cortex; MTG = middle temporal gyrus; OL = occipital lobe; OpPMF = operculopremotor fascicle; Pars Op = pars opercularis; Pars Orb = pars orbitalis; Pars Tr = pars triangularis; PCN = precuneus; PHG = parahippocampal gyrus; RH = right hemisphere; SLF-fp = frontoparietal segment of the superior longitudinal fascicle; SLF-tp = temporoparietal segment of the superior longitudinal fascicle; SMA = supplementary motor area; SMG = supramarginal gyrus; SPL = superior parietal lobule; STG = superior temporal gyrus; SubG = subgenual cortex; T-O = temporo-occipital cortex; TOF = temporo-occipital fascicle; TP = temporal pole; UF = uncinate fascicle; UNC = uncus; vPMC = ventral premotor cortex; WM = white matter.

**Table 4:** Connection patterns of white matter fascicles.

WM fascicles	Left Hemisphere		Right Hemisphere	
	Connectivity pattern	% (number)	Connectivity pattern	% (number)
**AF**	Op, vPMC, STG, MTG	0.35 (7)	vPMC, STG, MTG	0.40 (8)
	Tr, Op, vPMC, STG, MTG	0.30 (6)	OP, vPMC, STG, MTG	0.25 (5)
	MFG, Op, vPMC, STG, MTG	0.30 (6)	Op, vPMC, MTG	0.10 (2)
**SLF-fp**	vPMC, SMG	0.60 (12)	vPMC, SMG	0.60 (12)
	Op, vPMC, SMG	0.35 (7)	Op, vPMC, SMG	0.35 (7)
	Op, Tr, SMG	0.05 (1)	Op, Tr, SMG	0.05 (1)
**SLF-tp**	AG, SMG, STG, MTG	0.45 (9)	AG, SMG, STG, MTG	0.35 (7)
	AG, MTG	0.35 (7)	AG, STG, MTG	0.30 (6)
	AG, STG, MTG	0.20 (4)	AG, MTG	0.15 (3)
**UF**	FP, OrbF, SubG, Orb, UNC, PHG, TP	0.25 (5)	FP, OrbF, SubG, UNC, PHG, TP	0.20 (4)
	FP, OrbF, SubG, UNC, PHG, TP	0.15 (3)	OrbF, UNC, PHG, TP	0.15 (3)
	OrbF, UNC, PHG	0.10 (2)	FP, OrbF, SubG, Orb, UNC, PHG, TP	0.10 (2)
**TOF**	OL, T-O, ITG, MTG, TP	1.0 (20)	OL, T-O, ITG, MTG, TP	1.0 (20)
**IFOF**	MFG, FP, OrbF, Orb, Tr, MTG, ITG, T-O, OL	0.40 (8)	FP, OrbF, Orb, SPL, PCN, MTG, ITG, T-O, OL	0.15 (3)
	MFG, FP, OrbF, Orb, MTG, ITG, T-O, OL	0.20 (4)	FP, OrbF, Orb, PCN, MTG, ITG, T-O, OL	0.15 (3)
	MFG, FP, OrbF, Orb, Tr, PCN, MTG, ITG, T-O, OL	0.10 (2)	FP, OrbF, Orb, MTG, ITG, T-O, OL	0.10 (2)
**MdLF**	TP, STG, AG	0.35 (7)	TP, STG, SPL	0.30 (6)
	TP, STG, AG, OL	0.30 (6)	TP, STG, AG	0.15 (3)
	TP, STG, AG, SPL	0.15 (3)	TP, STG, AG, SPL	0.10 (2)
**FAF**	SMA, Tr, Op	0.95 (19)	SMA, Tr, Op	0.95 (19)
	SMA, Op	0.05 (1)	SMA, Op	0.05 (1)
**OpPMF**	Op, vPMC	1.0 (20)	Op, vPMC	1.0 (20)

Table giving the three most frequent occurrences (percentage; number in brackets) of connection patterns for each of the eight white matter fascicles (20 heathy subjects). AF = arcuate fascicle; AG = angular gyrus; FAF = frontal aslant fascicle; FP = frontal pole; IFOF = inferior fronto-occipital fascicle; ITG = inferior temporal gyrus; OrbF = orbitofrontal cortex; MdLF = middle longitudinal fascicle; MFG = middle frontal gyrus; MTG = middle temporal gyrus; OL = occipital lobe; Op = pars opercularis; OpPMF = operculopremotor fascicle; Orb = pars orbitalis; PCN = precuneus; PHG = parahippocampal gyrus; SLF-fp = frontoparietal segment of the superior longitudinal fascicle; SLF-tp = temporoparietal segment of the superior longitudinal fascicle; SMA = supplementary motor area; SMG = supramarginal gyrus; SPL = superior parietal lobule; STG = superior temporal gyrus; SubG = subgenual cortex; T-O = temporo-occipital cortex; TOF = temporo-occipital fascicle; TP = temporal pole; Tr = pars triangularis; UF = uncinate fascicle; UNC = uncus; vPMC = ventral premotor cortex; WM = white matter.

### Connections between WM Fascicles and BOLD Clusters

**Fig 5:**
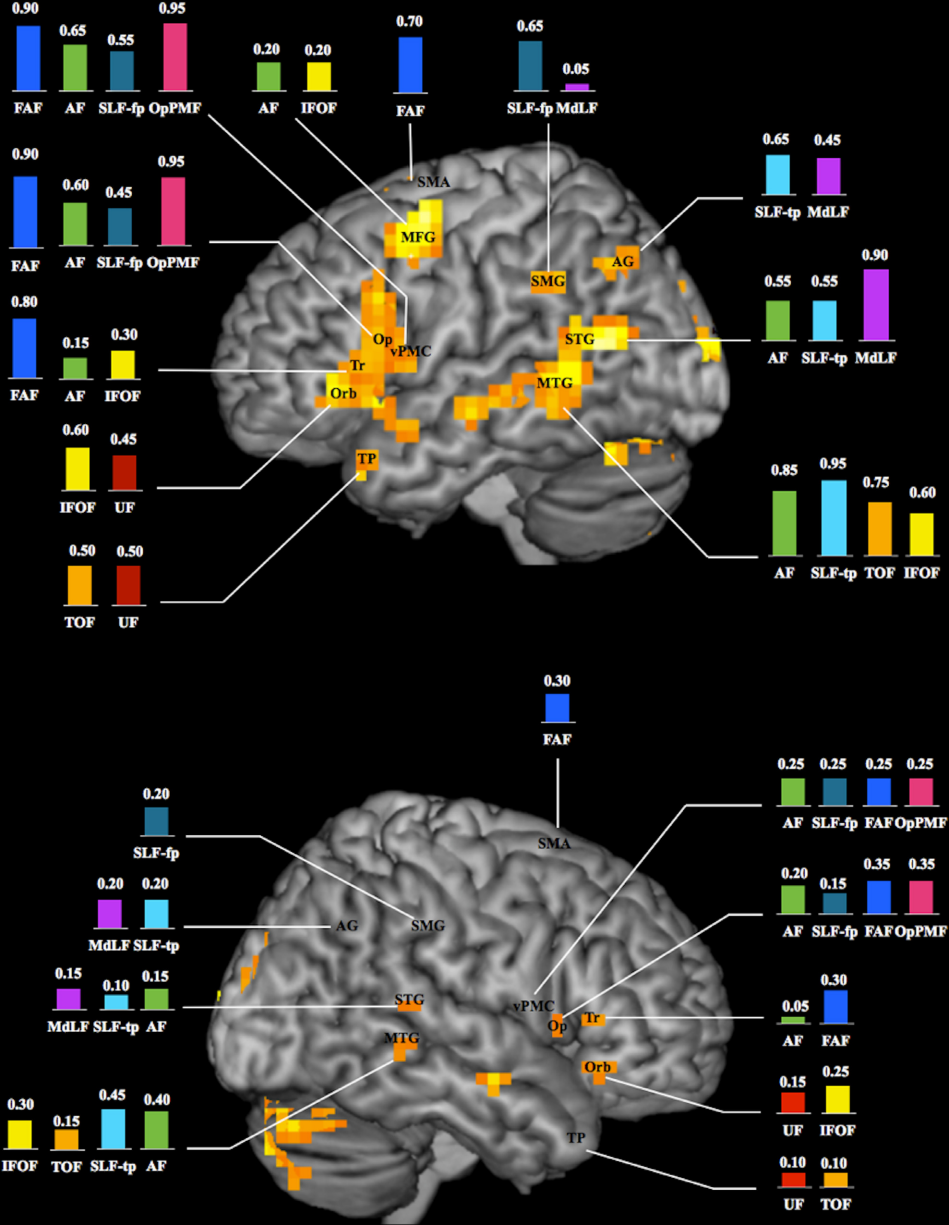
Schematic illustration of the language connectome. Occurrence (expressed in percentage; n = 20 healthy subjects) of connections between white matter fascicles and BOLD clusters within known essential language areas of the left (top) and right (bottom) hemispheres (3D renderings of fMRI group analysis; activations observed at individual level within the temporal pole, inferior parietal lobule and ventral premotor cortex of the right hemisphere are not shown; see text for details). AF = arcuate fascicle; AG = angular gyrus; FAF = frontal aslant fascicle; IFOF = inferior fronto-occipital fascicle; MdLF = middle longitudinal fascicle; MFG = middle frontal gyrus; MTG = middle temporal gyrus; Op = pars opercularis; OpPMF = operculopremotor fascicle; Orb = pars orbitalis; SLF-fp = frontoparietal segment of the superior longitudinal fascicle; SLF-tp = temporoparietal segment of the superior longitudinal fascicle; SMA = supplementary motor area; SMG = supramarginal gyrus; STG = superior temporal gyrus; TOF = temporo-occipital fascicle; TP = temporal pole; Tr = pars triangularis; UF = uncinate fascicle; vPMC = ventral premotor cortex.

The overall occurrences of WM fascicle–BOLD cluster connections within cortical territories known as essential language areas are reported in Fig 5 and S3 Table. As the analysis was performed at individual level, a number of WM fascicle–BOLD cluster connections were found within cortical territories of the right hemisphere where fMRI group analysis did not reveal areas of activations.

Within the left hemisphere, BOLD clusters in the most posterior part of Broca’s area (i.e. pars opercularis and triangularis of the IFG, BA 44/45) and vPMC (BA 6) were connected with BOLD clusters of the SMA through the FAF, and to Wernicke’s area (BA 21/22) through direct (i.e. AF) and indirect (i.e. SLF-fp + SLF-tp) dorsal pathways. The posterior part of Broca’s area was further connected to BOLD clusters in vPMC through the OpPMF. The anterior part of Broca’s area (i.e. pars orbitalis of the IFG, BA 47) was connected to Wernicke’s area by a ventral pathway composed of a direct (i.e. IFOF) and indirect segment (i.e. TOF + UF), with a relay at the level of the TP (BA 38). Wernicke’s area and Geschwind’s area (AG, BA 39) were connected through the MdLF.

### Correlation between Lateralization Indexes of WM Fascicles and BOLD Clusters

We found a global leftward lateralization of BOLD clusters (LI = 0.71±0.25) confirming the left-hemispheric language dominance of the 20 right-handed, healthy male subjects. This leftward functional lateralization of BOLD clusters was correlated with the leftward structural lateralization of AF volume (r = 0.78; p = 0.005).

## Discussion

DTI-FT in combination with fMRI enabled us to delineate the cortical terminations of eight language-related WM fascicles within the frontal, parietal, temporal and occipital lobes, in 20 healthy right-handed male subjects, and thus reveal further specific connectivity in the right and left hemispheres. Three important findings emerge: (1) the structural WM organization of the language network differs between the two hemispheres; (2) the bilateral variability of WM fascicle connection patterns underlines the high degree of anatomical variability among the normal population; (3) the cortical connections of language-related WM fascicles extend beyond the classical language areas revealed by fMRI, which may reflect an involvement in several distinctive functional brain processes.

DTI-FT carries several well-known technical biases that need to be considered when interpreting the results of this study: (1) very small fiber bundles were not identifiable due to the size of the voxels (1×1×3.5 mm^3^); (2) convergences of WM fascicles, e.g. IFOF and UF in the frontobasal lobe or IFOF and TOF in the temporobasal lobe, made it difficult to get a fined-grained distinction between neighboring fibers (despite optimization by manual placement of seeders); (3) FT within the gray–white matter interface was limited by the low anisotropy of this complex region. These limitations may have influenced our observations regarding the variability of cortical terminations of WM fascicles, especially those less frequently observed. Consequently, we focus here on the most robust and frequent results.

### Hemispheric Asymmetries in WM Connectivity

Asymmetric distribution of cognitive functions between the two hemispheres is a striking feature of the human brain, generally referred to as functional lateralization. The most prominent evidence for left-hemisphere specialization for language comes from studies of patients with aphasia secondary to stroke: aphasic right-handed adults almost invariably have lesions located in the left hemisphere [1]. Similar prevalence data have been found using fMRI, with left-hemisphere dominance for language in 95% of right-handed and 70% of left-handed individuals [34]. The structural correlates of language lateralization, however, are less clear. One of the major approaches to explain the ontogenesis of language lateralization is to premise that it reflects underlying structural hemispheric asymmetries. Studies have found that the planum temporale, a cortical area of the temporal lobe that largely overlaps into the classical Wernicke’s territory, is more developed in the left hemisphere than in the right [35,36]. However, the leftward asymmetry of the planum temporale does not correlate with lateralization of language functions as assessed by the intracarotid Sodium Amytal procedure [37]. In fact, leftward structural asymmetry of perisylvian WM looks a more likely anatomical substrate for language lateralization than cortical areas alone, especially given the significant correlation between the leftward functional lateralization of fMRI activations and the leftward structural lateralization of AF volume found here. Similar structure–function correlations have previously been reported with the demonstration of a direct relationship between degree of language lateralization and lateralization of the FA value [38,39] and number of streamlines of the AF [39]. Such structural asymmetries of AF may reflect differences in axonal diameters, myelination and fiber density at a microstructural level, again related to functional lateralization. Taken together, these lines of evidence point to the AF as a major structural connection underlying language functions. Furthermore, in accordance with previous studies [6,40,41], we found a significant asymmetry of the volumes of SLF-fp, SLF-tp and UF. Interestingly, these asymmetries appear to be balanced, with SLF-tp and UF being thicker in the left hemisphere and SLF-fp being thicker in the right. We cannot explain the rightward lateralization of SLF-fp, but the rightward distribution of certain subcomponents of language, such as prosody [42], may be one rationale.

A critical finding of our study is that language-related WM fascicles are also lateralized in terms of their connection patterns. For example, we observed striking inter-hemispheric differences in direct connections between Broca’s area and Wernicke’s area through the AF, with leftward lateralization in 60% of subjects and bilateral symmetrical distribution in only 40%. Preliminary findings from one study [43] showed that an asymmetric pattern of AF connections is associated with worse performance on a verbal memory task that relies on semantic association for retrieval (i.e. the California Verbal Learning Test). These findings support the notion that the lateralization of language to the left hemisphere is a striking feature of human brain organization, but paradoxically, a bilateral representation might ultimately be advantageous for certain language abilities. From a clinical point of view, it is tempting to hypothesize that this correlation between lateralization of WM fascicles and language performances participates in the varying degrees of aphasia severity and recovery potential following language-network lesions. We also demonstrated a striking lateralized pattern of connectivity for the MdLF and IFOF. The MdLF showed predominant connections between STG and AG on the left and STG and SPL on the right, as reported by Makris et al. [44] but not observed by others [45]. The IFOF showed prominent connections with SPL in the right hemisphere but not in the left. Future research would productively investigate the behavioral correlates of these asymmetries, which should be considered in the context of cerebral systems organization and lateralization of functions. In addition to the leftward lateralization for language, other lateralized functions such as spatial processing and attention in the SPL of the right hemisphere have also been reported [46]. A study investigating right-handed patients with spatial neglect secondary to right-hemisphere stroke has shown that a lesion to the IFOF may contribute to visual neglect in some cases [47].

### Inter-Subject Variability in WM Connectivity

In recent years, several teams have used DTI-FT to produce *in vivo* atlases of the major WM fascicles in the human brain, particularly those proposed to support language [11,30,31,48,49]. However, these atlases are built on the “average” anatomy in representative subjects, and few studies have addressed the inter-individual variability in the normal population, focusing on differences in the position and path of WM fascicles [50]. Here we found a high degree of inter-individual variations in patterns of anatomical connections. Although variability in position and path of WM fascicles can be explained by gross anatomical variability in brain size and shape, heterogeneity in connection patterns is a different story and may represent something more profound. Some individual variations may be related to inheritance while others may be the result, for instance, of normal aging, experiential learning, or development of new skills. Better knowledge of the anatomical variability in the normal population could help determine the spatial relationship between brain lesions and adjacent WM fascicles and thereby gain a deeper understanding of the mechanisms underpinning brain plasticity and recovery of functions [51].

### Functional Roles Inferred from Anatomical Connectivity

We assigned functionality to WM fascicles by analyzing their connections with BOLD clusters. We observed consistent connections between BOLD clusters and the eight WM fascicles, thus providing an extensive description of the language connectome. In addition, we demonstrated that fibers of language-related WM fascicles reach several other cortical territories involved in different functional brain networks, e.g. associative extrastriate cortex and T-O, which are involved in visual recognition and conceptualization; SPL and PCN (spatial attention and visuo-motor coordination); PHG (memory formation); prefrontal cortices (executive functions); OrbF (processing of emotional and behavioral aspects); SubG (mood). This distribution of fibers may reflect distinctive functional roles according to the cortical sites of termination. These data support the hypothesis that language-related WM fascicles are “multifunction” rather than specialized [19]. Future studies using specific tasks and implementing functional mapping (fMRI or magnetoencephalography) and structural mapping (such as DTI-FT) information are needed to further elucidate the role of these WM fascicles in human cognition.

## Conclusions

Combined DTI-FT and fMRI analysis was used to delineate and quantify eight language-related WM fascicles in 20 healthy adult male subjects, thus providing a detailed description of the WM language connectome. We found a leftward asymmetry of AF volume, reflecting the lateralization of language functions. Brain asymmetry extended up to hemispheric differences in patterns of anatomical connections, notably for the AF, IFOF and MdLF, which may relate to specific hemispheric abilities. The extensive cortical terminations observed reinforce the general evidence that the reported WM fascicles are by no means exclusively involved in language processing but likely support other cognitive skills.

## Supporting Information

S1 Glossary Terminology of WM fascicles. (DOCX)

S1 Table DTI-FT studies exploring the anatomical connectivity of language-related WM fascicles. a = anterior; AF = arcuate fascicle; AG = angular gyrus; ATL = anterior temporal lobe; BA = Brodmann area; d = dorsal; DLPFC = dorsolateral prefrontal cortex; EmC = extreme capsule; FAF = frontal aslant fascicle; FOP = frontal operculum; FP = frontal pole; IFOF = inferior fronto-occipital fascicle; ILF = inferior longitudinal fascicle; IPL = inferior parietal lobule; ITG = inferior temporal gyrus; m = middle; MdLF = middle longitudinal fascicle; MTG = middle temporal gyrus; OL = occipital lobe; OpPMF = operculopremotor fascicle; OrbF = orbitofrontal cortex; p = posterior; PCN = precuneus; PMC = premotor cortex; ROI = region of interest; SLF-fp = frontoparietal segment of the superior longitudinal fascicle; SLF-tp = temporoparietal segment of the superior longitudinal fascicle; SMG = supramarginal gyrus; SPL = superior parietal lobule; STG = superior temporal gyrus; TP = temporal pole; UF = uncinate fascicle; v = ventral; WM = white matter. * Seed region for fiber tracking.
(DOCX)

S2 Table Distribution of BOLD clusters (individual analysis). Occurrence (number; percentage in brackets) of BOLD clusters within cortical territories known to be essential language areas (20 healthy subjects). AG = angular gyrus; IFG = inferior frontal gyrus; LH = left hemisphere; MFG = middle frontal gyrus; MTG = middle temporal gyrus; RH = right hemisphere; SMA = supplementary motor area; SMG = supramarginal gyrus; STG = superior temporal gyrus; TP = temporal pole; vPMC = ventral premotor cortex.
(DOCX)

S3 Table Connections between WM fascicles and BOLD clusters. Occurrence (percentage; number in brackets) of connections between white matter fascicles and BOLD clusters within known essential language areas (20 healthy subjects). AF = arcuate fascicle; AG = angular gyrus; FAF = frontal aslant fascicle; FP = frontal pole; IFOF = inferior fronto-occipital fascicle; LH = left hemisphere; MdLF = middle longitudinal fascicle; MFG = middle frontal gyrus; OpPMF = operculopremotor fascicle; Pars Op = pars opercularis; Pars Orb = pars orbitalis; Pars Tr = pars triangularis; pMTG = posterior part of the middle temporal gyrus; pSTG = posterior part of the superior temporal gyrus; RH = right hemisphere; SLF-fp = frontoparietal segment of the superior longitudinal fascicle; SLF-tp = temporoparietal segment of the superior longitudinal fascicle; SMA = supplementary motor area; SMG = supramarginal gyrus; TOF = temporo-occipital fascicle; TP = temporal pole; UF = uncinate fascicle; vPMC = ventral premotor cortex; WM = white matter.
(DOCX)

S1 Text Anatomical connectivity findings. (DOCX)
