# Supplementary material for: Combined DTI Tractography and Functional MRI Study of the Language Connectome in Healthy Volunteers: Extensive Mapping of White Matter Fascicles and Cortical Activations
Source: PLoS One. 2016 Mar 30;11(3):e0152614. doi: 10.1371/journal.pone.0152614 (PMC4814138; doi:10.1371/journal.pone.0152614)
Supplement: S1 Glossary — (DOCX) [file pone.0152614.s001.docx]

The sometimes confusing terminology of WM fascicles is revisited here to facilitate further analysis:

**Arcuate fascicle (AF).** The AF has also been called the arcuate fasciculus of Burdach, the superior frontal fascicle, or the fasciculus longitudinalis superior [1,2]. The AF directly connects the frontal and temporal lobes. It is located laterally to the superior part of the corona radiata, and arches around the insula, from where it gains its typical curved shape and name. Catani et al. [3] also described a parallel fiber tract between the inferior frontal cortex and IPL (“Geschwind area”) and a fiber tract between the IPL and the posterior temporal cortex. The authors referred to these two tracts as the indirect AF, but in subsequent studies (see below) these indirect pathways that pass through the parietal lobe have been added to the superior longitudinal fascicle (SLF).

**Superior longitudinal fascicle (SLF).** The AF and SLF were first regarded as a single bundle in humans [2]. However, subsequent investigations in the monkey brain dissociated the AF from the SLF [4]: the AF has been assigned to the fiber bundle that connects the frontal and temporal lobes directly without sending out branches to the parietal lobe, whereas the fibers connecting the frontal and parietal cortex have been assigned to the SLF. The SLF has been further divided into three separate frontoparietal bundles, named SLF I, II and III, according to their respective cortical sites of termination. SLF I has been described as running medially, connecting the superior frontal gyrus with the SPL; SLF II has been described as travelling from the dorsal premotor cortex and dorsolateral prefrontal cortex to the caudal IPL; SLF III has been described as linking vPMC to the rostral IPL. The same subdivision and connectivity profiles of these three fiber bundles have been identified in humans [5]. Moreover, a fourth fiber tract has been delineated that consists of long and short arched fibers that connect the posterior temporal cortex with the IPL. As stated earlier, this temporoparietal tract was first described as an additional component of the AF [3], but with the adaptation of the monkey nomenclature to humans, it has subsequently been associated to the SLF III [6], which is therefore actually composed of two separate segments running parallel and lateral to the AF: an anterior or horizontal or frontoparietal segment (SLF-fp), and a posterior or vertical or temporoparietal segment (SLF-tp).

**Uncinate fascicle (UF).** The UF connects the basal frontal lobe with the anterior temporal lobe. It is located just beneath the IFOF at the level of the anterior floor of the extreme capsule.

**Temporo-occipital fascicle (TOF).** The TOF has also been called the fasciculus longitudinalis inferior [2]. It connects the occipital lobe with the temporal lobe, running laterally to the IFOF. The inferior longitudinal fascicle of monkeys [4] broadly includes the fibers of the human TOF as well as a set of temporoparietal fibers that may correspond to the human SLF-tp described above.

**Inferior fronto-occipital fascicle (IFOF).** The IFOF connects the basal frontal lobe with the temporo-occipito-parietal region. The fibers of the IFOF run adjacent to the TOF for much of their trajectory through the occipital and posterior temporal regions, before converging at the level of the extreme capsule.

**Extreme capsule (EmC).** This nomenclature is used in monkey anatomy [4] and corresponds to the system of fibers that run between claustrum and insula and link the temporal and frontal lobes. Our view, backed up by others [7,8], is that this system is conceptually the same as the IFOF.

**Middle longitudinal fascicle (MdLF).** Initially described in the monkey, the MdLF has also been delineated in the human brain [9], where it runs from the TP to the caudal end of the superior temporal gyrus and further extends dorsally and caudally within the IPL.

**Frontal aslant fascicle (FAF).** The FAF has recently been described as an oblique bundle that connects the most posterior part of Broca’s area (i.e. pars opercularis of the IFG) and vPMC to the SMA and pre-SMA in the superior frontal gyrus [10].

**Operculopremotor fascicle (OpPMF).** The existence of the OpPMF in the human brain has only recently been demonstrated [11]. It is composed of well-organized U-shaped fibers connecting the pars opercularis of the IFG with vPMC.

**References**

1. Riley H. An atlas of the basal ganglia, brain stem and spinal cord. Baltimore: Williams & Wilkins; 1953.

2. Déjerine JJ. Anatomie des centres nerveux. Vol. 1 Paris: Rueff et Cie; 1895.

3. Catani M, Jones DK, Ffytche DH. Perisylvian language networks of the human brain. Ann Neurol. 2005;57: 8-16.

4. Schmahmann J, Pandya D. Fiber pathways of the brain. New York: Oxford University Press, Inc; 2006.

5. Frey S, Campbell JSW, Pike GB, Petrides M. Dissociating the human language pathways with high angular resolution diffusion fiber tractography. J Neurosci 2008;28: 11435-11444.

6. Galantucci S, Tartaglia MC, Wilson SM, Henry ML, Filippi M, Agosta F, et al. White matter damage in primary progressive aphasias: a diffusion tensor tractography study. Brain 2011; 134: 3011-3029.

7. Parker GJ, Luzzi S, Alexander DC, Wheeler-Kingshott CA, Ciccarelli O, Lambon Ralph MA. Lateralization of ventral and dorsal auditory-language pathways in human brain. Neuroimage. 2005;24: 656-666.

8. Duffau H. A re-examination of neural basis of language processing: proposal of a dynamic hodotopical model from data provided by brain stimulation mapping during picture naming. Brain Lang 2014;131: 1-10.

9. Makris N, Papadimitriou GM, Kaiser JR, Sorg S, Kennedy DN, Pandya DN. Delineation of the middle longitudinal fascicle in humans: a quantitative, in vivo, DT-MRI study. Cereb Cortex. 2009;19: 777-785.

10. Catani M, Dell’Acqua F, Vergani F, Malik F, Hodge H, Roy P, et al. Short frontal lobe connections in the human brain. Cortex. 2012;48: 273-291.

11. Lemaire JJ, Golby A, Wells WM 3^rd^, Pujol S, Tie Y, Rigolo L, et al. Extended Broca’s area in the functional connectome of language in adults: combined cortical and subcortical single-subject analysis using fMRI and DTI tractography. Brain Topogr. 2013;26: 428-441.
