# Supplementary material for: Combined DTI Tractography and Functional MRI Study of the Language Connectome in Healthy Volunteers: Extensive Mapping of White Matter Fascicles and Cortical Activations
Source: PLoS One. 2016 Mar 30;11(3):e0152614. doi: 10.1371/journal.pone.0152614 (PMC4814138; doi:10.1371/journal.pone.0152614)
Supplement: S1 Table — a = anterior; AF = arcuate fascicle; AG = angular gyrus; ATL = anterior temporal lobe; BA = Brodmann area; d = dorsal; DLPFC = dorsolateral prefrontal cortex; EmC = extreme capsule; FAF = frontal aslant fascicle; FOP = frontal operculum; FP = frontal pole; IFOF = inferior fronto-occipital fascicle; ILF = inferior longitudinal fascicle; IPL = inferior parietal lobule; ITG = inferior temporal gyrus; m = middle; MdLF = middle longitudinal fascicle; MTG = middle temporal gyrus; OL = occipital lobe; OpPMF = operculopremotor fascicle; OrbF = orbitofrontal cortex; p = posterior; PCN = precuneus; PMC = premotor cortex; ROI = region of interest; SLF-fp = frontoparietal segment of the superior longitudinal fascicle; SLF-tp = temporoparietal segment of the superior longitudinal fascicle; SMG = supramarginal gyrus; SPL = superior parietal lobule; STG = superior temporal gyrus; TP = temporal pole; UF = uncinate fascicle; v = ventral; WM = white matter. * Seed region for fiber tracking. (DOCX) [file pone.0152614.s002.docx]

| **Study** | **Method** | **WM fascicle nomenclature assigned by the authors** | **Distant areas connected** | |
| --- | --- | --- | --- | --- |
| Catani et al. (2005) [1] | DTI tractography with single-ROI approach, or double-ROI approach for partitioning (11 healthy volunteers) | AF, direct pathway | “Broca’s territory” (BA 44) | “Wernicke’s territory” (pSTG/MTG) |
|  |  | AF, indirect pathway, anterior segment | “Broca’s territory” (vPMC) | “Geschwind’s territory” (IPL) |
|  |  | AF, indirect pathway, posterior segment | “Geschwind’s territory” (IPL) | “Wernicke’s territory” (pSTG/MTG) |
| Parker et al. (2005) [2] | DTI tractography with double-ROI approach (11 healthy volunteers) | AF | BA 44* | pSTG*, MTG |
|  |  | UF or superior part of EmC | BA 44* | ATL, STG* |
| Friederici et al. (2006) [3] | DTI tractography using single-ROI approach and fMRI for seeding (40 healthy volunteers) | AF | BA 44* | m/pSTG |
|  |  | UF | FOP* | ATL |
| Anwander et al. (2007) [4] | DTI tractography with single-ROI approach (6 healthy volunteers) | AF/SLF | BA 44/45*, vPMC | Parietal cortex, temporal cortex |
|  |  | UF | FOP* | ATL |
|  |  | IFOF and ILF | BA 45* | Posterior temporal cortex |
| Saur et al. (2008) [5] | DTI tractography using multi-ROI approach and fMRI for seeding (33 healthy volunteers) | AF/SLF III | Pars opercularis (BA 44)*, dPMC (BA 6)* | pSTG* |
|  |  | EmC | FOP*, Pars orbitalis and triangularis* | Fusiform gyrus*, a/pMTG*, a/pSTG* |
| Frey et al. (2008) [6] | DTI tractography with double-ROI approach (12 healthy volunteers) | AF | Pars opercularis (BA 44)*, dPMC (BA 6)*, BA 8 | pSTG* |
|  |  | SLF III | BA 44* | SMG* |
| Glasser and Rilling (2008) [7] | DTI tractography with double-ROI approach (20 healthy volunteers) | AF | BA 6/9/44/45 | pSTG (BA 22), pMTG (BA21/37) |
| Hua et al. (2009) [8] | DTI tractography with multi-ROI approach (28 healthy volunteers) | UF | FP (BA 10) | TP (BA 38) |
|  |  | ILF | TP (BA 38) | OL (BA 18/19) |
|  |  | IFOF | OL (BA 18/19) | FP (BA 10) |
| Makris et al. (2009) [9] | DTI tractography with single-ROI approach (4 heathy volunteers) | MdLF | STG | AG |
| Martino et al. (2011) [10] | DTI tractography with multi-ROI approach (1 heathy volunteer), compared with postmortem fiber dissection (30 hemispheres) | AF | pFOP | pMTG/ITG |
|  |  | SLF, horizontal segment | pFOP | SMG, pSTG |
|  |  | SLF, vertical segment | AG | pMTG |
|  |  | UF | Medial and lateral OrbF | TP, aSTG, aMTG |
|  |  | IFOF | Frontal lobe | OL, SPL, temporobasal cortex |
|  |  | ILF | TP | OL, fusiform gyrus |
| Sarubbo et al. (2011) [11] | DTI tractography with double- and multi-ROI approaches (1 healthy volunteer), compared with postmortem fiber dissection (10 hemispheres) | IFOF | Pars triangularis and orbitalis, DLPFC, OrbF, FP | pMTG, SPL, OL (BA 18/19), fusiform gyrus |
| Catani et al. (2012) [12] | DTI tractography (1 healthy volunteer) with double-ROI approach, compared with postmortem fiber dissection (1 hemisphere) | FAF | SMA and pre-SMA | Pars opercularis and triangularis, vPMC |
| Thiebaut de Schotten et al. (2012) [13] | DTI tractography with double and multi-ROI approaches (1 healthy volunteer) | AF | IFG (BA 44/45), vPMC (BA 6), pMFG (BA 9) | pSTG (BA 22/41/42), pMTG/ITG (BA 21/37) |
|  |  | IFOF | OrbF (BA 11), FP (BA 10), DLPFC (BA 9) | OL (BA 18/19) |
| Makris et al. (2013) [14] | DTI tractography with multi-ROI approach (39 healthy volunteers) | MdLF | TP, STG* | AG, SMG, SPL, PCN, OL |
| Menjot de Champfleur et al. (2013) [15] | DTI tractography with multi-ROI approach (4 healthy volunteers) | MdLF | STG | AG, TP |
| Lemaire et al. (2013) [16] | DTI tractography with multi-ROI approach (12 healthy volunteers) | AF | Pars triangularis and opercularis, vPMC | STG/MTG |
|  |  | SLF-fp | Pars opercularis, vPMC | “Geschwind’s area” |
|  |  | OpPMF | Pars opercularis | vPMC |

**References**

1. Catani M, Jones DK, Ffytche DH. Perisylvian language networks of the human brain. Ann Neurol. 2005;57: 8-16.

2. Parker GJ, Luzzi S, Alexander DC, Wheeler-Kingshott CA, Ciccarelli O, Lambon Ralph MA. Lateralization of ventral and dorsal auditory-language pathways in human brain. Neuroimage. 2005;24: 656-666.

3. Friederici AD, Bahlmann J, Heim S, Schubotz RI, Anwander A. The brain differentiates human and non-human grammars: functional localization and structural connectivity. Proc Natl Acad Sci USA. 2006;103: 2458-2463.

4. Anwander A, Tittgemeyer M, von Cramon DY, Friederici AD, Knösche TR. Connectivity-based parcellation of Broca’s area. Cereb Cortex. 2007;17: 816-825.

5. Saur D, Kreher BJ, Schnell S, Kümmerer D, Kellmeyer P, Vry MS, et al. Ventral and dorsal pathways for language. PNAS. 2008;105: 18035-18040.

6. Frey S, Campbell JSW, Pike GB, Petrides M. Dissociating the human language pathways with high angular resolution diffusion fiber tractography. J Neurosci 2008;28: 11435-11444.

7. Glasser MF, Rilling, JK. DTI tractography of the human brain’s language pathways. Cereb Cortex. 2008;18: 2471-2482.

8. Hua K, Oishi K, Zhang J, Wakana S, Yoshioka T, Zhang W, et al. Mapping of functional areas in the human cortex based on connectivity through association fibers. Cereb Cortex. 2009;19: 1889-1895.

9. Makris N, Papadimitriou GM, Kaiser JR, Sorg S, Kennedy DN, Pandya DN. Delineation of the middle longitudinal fascicle in humans: a quantitative, in vivo, DT-MRI study. Cereb Cortex. 2009;19: 777-785.

10. Martino J, De Witt Hamer PC, Vergani F, Brogna C, de Lucas EM, Vazquez-Barquero A, et al. Cortex-sparing fiber dissection: an improved method for the study of white matter anatomy of the human brain. J Anat. 2011;219: 531-541.

11. Sarubbo S, De Benedictis A, Maldonado IL, Basso G, Duffau H. Frontal terminations for the inferior fronto-occipital fascicle: anatomical dissection, DTI study and functional considerations on a multi-component bundle. Brain Struct Funct. 2013;218: 21-37.

12. Catani M, Dell’Acqua F, Vergani F, Malik F, Hodge H, Roy P, et al. Short frontal lobe connections in the human brain. Cortex. 2012;48: 273-291.

13. Thiebaut de Schotten M, Dell’Acqua F, Valabregue R, Catani M. Monkey to human comparative anatomy of the frontal lobe association tracts. Cortex. 2012;48: 82-96.

14. Makris N, Preti MG, Asami T, Pelavin P, Campbell B, Papadimitriou GM, et al. Human middle longitudinal fascicle: variations in patterns of anatomical connections. Brain Struct Funct. 2013;218: 951-968.

15. Menjot de Champfleur N, Maldonado IL, Moritz-Gasser S, Machi P, Le Bars E, Bonafé A et al. Middle longitudinal fasciculus delineation within language pathways: a diffusion tensor imaging study in human. Eur J Radiol. 2013;82: 151-157.

16. Lemaire JJ, Golby A, Wells WM 3^rd^, Pujol S, Tie Y, Rigolo L, et al. Extended Broca’s area in the functional connectome of language in adults: combined cortical and subcortical single-subject analysis using fMRI and DTI tractography. Brain Topogr. 2013;26: 428-441.
