# Supplementary material for: Combined DTI Tractography and Functional MRI Study of the Language Connectome in Healthy Volunteers: Extensive Mapping of White Matter Fascicles and Cortical Activations
Source: PLoS One. 2016 Mar 30;11(3):e0152614. doi: 10.1371/journal.pone.0152614 (PMC4814138; doi:10.1371/journal.pone.0152614)
Supplement: S2 Table — Occurrence (number; percentage in brackets) of BOLD clusters within cortical territories known to be essential language areas (20 healthy subjects). AG = angular gyrus; IFG = inferior frontal gyrus; LH = left hemisphere; MFG = middle frontal gyrus; MTG = middle temporal gyrus; RH = right hemisphere; SMA = supplementary motor area; SMG = supramarginal gyrus; STG = superior temporal gyrus; TP = temporal pole; vPMC = ventral premotor cortex. (DOCX) [file pone.0152614.s003.docx]

| **Cortical territories known as essential language areas** | | **Occurrence of BOLD clusters** | |
| --- | --- | --- | --- |
|  |  | **LH** | **RH** |
| **Frontal lobe** | **IFG, pars orbitalis** | 14 (0.70) | 5 (0.25) |
|  | **IFG, pars triangularis** | 18 (0.90) | 6 (0.30) |
|  | **IFG, pars opercularis** | 19 (0.95) | 7 (0.35) |
|  | **MFG** | 12 (0.60) | 7 (0.35) |
|  | **SMA** | 14 (0.70) | 6 (0.30) |
|  | **vPMC** | 19 (0.95) | 5 (0.25) |
| **Temporal lobe** | **TP** | 14 (0.70) | 2 (0.10) |
|  | **STG** | 20 (1.0) | 3 (0.15) |
|  | **MTG** | 20 (1.0) | 10 (0.50) |
| **Parietal lobe** | **SMG** | 13 (0.65) | 4 (0.20) |
|  | **AG** | 16 (0.80) | 8 (0.40) |
