# Supplementary material for: Combined DTI Tractography and Functional MRI Study of the Language Connectome in Healthy Volunteers: Extensive Mapping of White Matter Fascicles and Cortical Activations
Source: PLoS One. 2016 Mar 30;11(3):e0152614. doi: 10.1371/journal.pone.0152614 (PMC4814138; doi:10.1371/journal.pone.0152614)
Supplement: S3 Table — Occurrence (percentage; number in brackets) of connections between white matter fascicles and BOLD clusters within known essential language areas (20 healthy subjects). AF = arcuate fascicle; AG = angular gyrus; FAF = frontal aslant fascicle; FP = frontal pole; IFOF = inferior fronto-occipital fascicle; LH = left hemisphere; MdLF = middle longitudinal fascicle; MFG = middle frontal gyrus; OpPMF = operculopremotor fascicle; Pars Op = pars opercularis; Pars Orb = pars orbitalis; Pars Tr = pars triangularis; pMTG = posterior part of the middle temporal gyrus; pSTG = posterior part of the superior temporal gyrus; RH = right hemisphere; SLF-fp = frontoparietal segment of the superior longitudinal fascicle; SLF-tp = temporoparietal segment of the superior longitudinal fascicle; SMA = supplementary motor area; SMG = supramarginal gyrus; TOF = temporo-occipital fascicle; TP = temporal pole; UF = uncinate fascicle; vPMC = ventral premotor cortex; WM = white matter. (DOCX) [file pone.0152614.s004.docx]

| **BOLD cluster in known language areas** | **AF** | | **SLF-fp** | | **SLF-tp** | | **UF** | | **TOF** | | **IFOF** | | **MdLF** | | **FAF** | | **OpPMF** | |
| --- | --- | --- | --- | --- | --- | --- | --- | --- | --- | --- | --- | --- | --- | --- | --- | --- | --- | --- |
|  | **LH** | **RH** | **LH** | **RH** | **LH** | **RH** | **LH** | **RH** | **LH** | **RH** | **LH** | **RH** | **LH** | **RH** | **LH** | **RH** | **LH** | **RH** |
| **SMA** | — | — | — | — | — | — | — | — | — | — | — | — | — | — | 0.70 (14) | 0.30 (6) | — | — |
| **MFG** | 0.20 (4) | 0.05 (1) | — | — | — | — | — | — | — | — | 0.20 (4) | — | — | — | — | — | — | — |
| **Pars Orb** | — | — | — | — | — | — | 0.45 (9) | 0.15 (3) | — | — | 0.60 (12) | 0.25 (5) | — | — | — | — | — | — |
| **Pars Tr** | 0.15 (3) | 0.05 (1) | — | — | — | — | — | — | — | — | 0.30 (6) | — | — | — | 0.80 (16) | 0.30 (6) | — | — |
| **Pars Op** | 0.60 (12) | 0.20 (4) | 0.45 (9) | 0.15 (3) | — | — | — | — | — | — | — | — | — | — | 0.90 (18) | 0.35 (7) | 0.95 (19) | 0.35 (7) |
| **vPMC** | 0.65 (13) | 0.25 (5) | 0.55 (11) | 0.25 (5) | — | — | — | — | — | — | — | — | — | — | 0.90 (18) | 0.25 (5) | 0.95 (19) | 0.25 (5) |
| **SMG** | — | — | 0.65 (13) | 0.20 (4) | — | — | — | — | — | — | — | — | 0.05 (1) | — | — | — | — | — |
| **AG** | — | — | — | — | 0.65 (13) | 0.20 (4) | — | — | — | — | — | — | 0.45 (9) | 0.20 (4) | — | — | — | — |
| **pSTG** | 0.55 (11) | 0.15 (3) | — | — | 0.55 (11) | 0.10 (2) | — | — | — | — | — | — | 0.90 (18) | 0.15 (3) | — | — | — | — |
| **pMTG** | 0.85 (17) | 0.40 (8) | — | — | 0.95 (19) | 0.45 (9) | — | — | 0.75 (15) | 0.15 (3) | 0.60 (12) | 0.30 (6) | — | — | — | — | — | — |
| **TP** | — | — | — | — | — | — | 0.50 (10) | 0.10 (2) | 0.50 (10) | 0.10 (2) | — | — | — | — | — | — | — | — |
