# Supplementary material for: Combined DTI Tractography and Functional MRI Study of the Language Connectome in Healthy Volunteers: Extensive Mapping of White Matter Fascicles and Cortical Activations
Source: PLoS One. 2016 Mar 30;11(3):e0152614. doi: 10.1371/journal.pone.0152614 (PMC4814138; doi:10.1371/journal.pone.0152614)
Supplement: S1 Text — (DOCX) [file pone.0152614.s005.docx]

The following sections detail the anatomical connectivity of the eight WM fascicles systematically reconstructed within the left and right hemispheres (also see Tables 3 and 4):

**Arcuate fascicle (AF).** The AF (direct Broca-Wernicke pathway) connected with pMTG/pSTG (BA 21/22), vPMC (BA 6), pars opercularis and triangularis of the IFG (BA 44/45), and posterior part of the MFG (BA 9). The anatomical connectivity of AF showed pronounced hemispheric differences, with predominant direct connections between Broca’s area and Wernicke’s area in the left hemisphere, but not in the right.

**Superior longitudinal fascicle part III (SLF III).** In contrast to the AF, the SLF III (indirect Broca-Wernicke pathway) showed a relatively symmetrical connection pattern between the left and right hemispheres, with SLF-fp connecting SMG (BA 40), vPMC and pars opercularis of the IFG, and SLF-tp mainly connecting AG (BA 39) and pMTG/pSTG.

**Uncinate fascicle (UF).** The fibers of the UF originated from the TP (BA 38), UNC (BA 34) and PHG (BA 28). After a U-turn, the fibers of the UF then passed through the anterior floor of the extreme capsule, immediately ventral to the IFOF, before entering the frontal lobe. As it entered the frontal lobe, the UF divided into two layers. The first layer was superficial and laterally-oriented, terminating mainly in the pars orbitalis of the IFG (BA 47) and lOrbF (BA 11). The second layer was deeper and consisted of an anterior branch continuing toward the FP (BA 10) and mOrbF (BA 12) and a posteromedial branch ending in the SubG (BA 25). We were able to observe several connectivity profiles of UF connecting subsets of the eight cortical territories, but without finding any notable hemispheric asymmetry.

**Temporo-occipital fascicle (TOF).** Tractographic reconstructions of the TOF showed constant cortical connectivity across hemispheres, with fibers arising in the OL (BA 18/19) and T-O (BA 37) then projecting to the ITG (BA 20), MTG (BA 21) and TP (BA 38).

**Inferior fronto-occipital fascicle (IFOF).** Posteriorly, the IFOF originated mainly from the OL (extrastriate cortex) including cuneus and lateral occipital gyri (BA 18/19), T-O including fusiform gyrus (BA 37), pITG/pMTG (BA 20/21), PCN (BA 7) and SPL (BA 7). The IFOF was differentiable from the TOF lining the IFOF within the occipital and posterior temporal lobes, along the lateral wall of lateral ventricle. At the junction of the temporal and frontal lobes, the IFOF narrowed as it passed through the extreme capsule, and then divided into two layers as it entered the frontal lobe. The first layer was superficial and oriented laterally, terminating mainly in the pars orbitalis of the IFG (BA 47) and lOrbF (BA 11). The second layer was deeper and featured two portions: a posterior branch directed dorsally to the MFG (BA 9/46) and an anterior branch directed ventrally to end in the mOrbF (BA 12) and FP (BA 10). The anatomical connectivity of the IFOF presented marked asymmetry between the two hemispheres due to prominent connections with SPL and PCN in the right hemisphere but not in the left.

**Middle longitudinal fascicle (MdLF).** The MdLF mainly connected with the TP (BA 38), STG (BA 22), AG (BA 39), SPL (BA 7), PCN (BA 7) and OL (BA 18/19). An important finding was that the MdLF showed a striking lateralized pattern, with connections predominantly between the TP, STG and AG in the left hemisphere and the TP, STG and SPL in the right.

**Frontal aslant fascicle (FAF).** In both hemispheres, the FAF connected the SMA (dorsal part of BA 6) with the most posterior part of Broca’s area (pars opercularis and triangularis of the IFG, BA 44/45) and vPMC (ventral part of BA 6).

**Operculopremotor fascicle (OpPMF).** The OpPMF comprised well-organized U-shapedfibers connecting the pars opercularis of the IFG (BA 44) with vPMC (BA 6), and mapped symmetrically between the two hemispheres.
